# Quality Criteria for Studies Assessing the Acute Effects of Heading: Results from a UEFA Expert Panel

**DOI:** 10.1007/s40279-023-01977-z

**Published:** 2023-12-27

**Authors:** Kerry Peek, Martino Franchi, Koen Lemmink, Paul Balsom, Tim Meyer

**Affiliations:** 1 Discipline of Physiotherapy, Sydney School of Health Sciences, Faculty of Medicine and Health, The University of Sydney, Sydney, NSW Australia; 2 Department of Biomedical Sciences, University of Padova, Padua, Italy; 3 Department of Human Movement Sciences, University Medical Centre Groningen, University of Groningen, Groningen, The Netherlands; 4 Swedish Football Association, Solna, Stockholm, Sweden; 5 Institute of Sports and Preventive Medicine, Saarland University, Saarland, Germany

## Abstract

There is increasing concern regarding the effects of heading in football on brain health including cognitive, behavioural and neuromotor function, with research suggesting an association between repeated ball-head impacts and neurodegenerative disease. While longitudinal studies to determine the long-term consequences of heading are challenging, there have been short-term ‘acute’ studies conducted, with some studies lacking appropriate methodology to ensure valid results. The Union of European Football Associations (UEFA) established a panel of experts to determine methodological recommendations for the conduct of studies that explore the acute effects of heading (defined as a single session of heading conducted either in a laboratory setting or following match play or a training session). The aim of this panel was to create quality criteria for acute heading studies that will form part of the eligibility assessment when applying for UEFA research funding (although the criteria can be applied to the conduct of acute heading research more widely). This process was deemed necessary to counter studies with poor methodological quality that used heading trials that did not accurately represent player exposure to ball-head impacts through football practice and match play (such as small sample sizes, unrealistically high heading exposure, and a lack of consideration of confounding variables). The panel identified core design decisions that authors should consider when designing and conducting acute heading research, with key methodological requirements for each domain pertaining to participants, heading trials, confounding variables, statistics and dependent/target variables and their measurement. After two rounds of reviews, the final list of quality criteria was agreed by the panel and will be applied to the next round of UEFA grant applications.

**Supplementary Information:**

The online version contains supplementary material available at 10.1007/s40279-023-01977-z.

## Key Points

**Table:** 

There are concerns that studies exploring the acute effects of heading lack appropriate methodology to ensure valid results.
This Current Opinion article provides recommendations on the minimum quality criteria that should be considered when designing and conducting acute heading research studies specifically related to: participants, heading trials, confounding variables, statistics, and dependent/target variables and their measurement.

## Introduction

In recent years, an increasing number of scientific studies have been published regarding the effects of (repeated) heading on brain structure and function (including cognition) [1–3]. The need for these studies is in response to the reported association between heading and the development of neurodegenerative disease in later life [4–6]. Theoretically, such an effect might be caused by accumulated damage from all performed headers, football-related head injuries including concussion (most likely sustained during heading duels), a combination of both and/or other factors associated with the game of football. However, neurodegeneration is likely to develop only after a delay of many years or even decades, meaning that the degree of association between heading and the development of neurodegenerative disease remains unclear [2]. Given the difficulty in conducting longitudinal studies (or even cross-sectional studies that include heading or head injury prevalence data) to determine the long-term consequences of heading, many researchers have instead investigated the short-term (‘acute’) effects (i.e. after a single session of heading conducted either in a laboratory setting or following match play or a training session) [1]. The feasibility of such studies means that results can realistically be expected within a time frame more acceptable for researchers and football stakeholders, who are required to draft policies and guidelines based on the best available knowledge at that time. However, some methodological requirements should be considered by authors to ensure that the outcomes of their studies are valid.

The Union of European Football Associations (UEFA) has already developed and published recommendations for heading in youth football in 2020 as well as sponsored studies in this area, including the UEFA Heading Study [7]. In 2022, its medical committee nominated an ‘Expert Panel on Heading’ which formulated minimum methodological requirements for acute heading research as part of the eligibility criteria when applying for funding from UEFA. It was felt that minimum quality criteria were warranted based on published studies that either lacked external validity despite claiming to be designed to inform football practice [1] or failed to make a proper distinction between the effects of heading and head injuries (including concussion) [2].

## Examples of Studies with an Inappropriate Design

A number of studies have explored the acute effects of heading on various outcome measures, including cognitive function and biochemical changes [7–10]. However, they have employed laboratory-based heading trials that may not represent typical match play or training because of the high number of headers (e.g. 15–20 headers within one heading session) [8–10], the frequency at which headers occur (e.g. 20 headers within 3 min or one header every 9 s) [8] or the ball delivery method (including balls dropped from a height) [11]. These methodological decisions are important to note as data from a recent systematic review demonstrated that the mean number of headers per player per game ranged from 0 to 11 headers in boys (aged 11 years and older) and men, and from 0 to 7 headers in girls (aged 11 years and older) and women [2]. Considering that most matches are 70–90 min in duration (depending on the age group), these data suggest that the mean number of headers is conservatively estimated at one header per player every 6–10 min. While it is accepted that heading frequency will not be uniform across every playing position with many players completing a series of headers within a short amount of time, the speed, distance and delivery of these balls will also vary [12]. For instance, research has consistently shown that headers from long balls, particularly goal and corner kicks, demonstrate higher head impact magnitudes during heading (generally known as high-force or high-velocity headers) than balls from free play and throw-ins [12, 13]. Additionally, it is more likely that there will be longer time periods between headers from goal and corner kicks than during free play within any given match. It is also important that researchers use balls that are the appropriate size for the age group of participants as well as being inflated to ball pressures that conform to International Football Association Board ‘Laws of the Game.’ It is also important that researchers consider participants’ technical proficiency in the execution of headers as both technique and location of head contact (i.e. forehead, top of the head) can also influence head impact magnitudes during heading [14].

The speed of ball delivery also needs consideration to ensure that the speed (and angle of flight) replicate match scenarios. Studies on the acute effects of heading have used ball speeds varying from 21.6 to 88.5 km/h [15, 16]. While it is acknowledged that ball speeds vary considerably between ball delivery methods, the concern is that studies that deliver more than ten high-velocity balls within an unrealistically short period of time could be exposing research participants to unnecessary risk while at the same time not being representative of football match play. As a general observation, studies that employ ten or more headers delivered within a shorter period of time (i.e. one header every 30–60 s) are more likely to demonstrate acute changes in participants than studies using a lower number or more time between headers [1, 9, 17, 18].

Other methodological issues in earlier published acute heading studies include the study design and exertional level of comparator activities. Common study designs include cross-over designs (to demonstrate within-participant effects) as well as experimental designs with a separate control group of participants (to demonstrate between-participant effects) [1]. If experimental and control groups are included, participants should be matched for age, sex and athletic ability. In terms of the exertional level of comparator activities, the confounding effect of exercise and physical fatigue on results should be considered [1]. Therefore, the control or comparator condition needs to be matched in exertional demands to the heading trials, with exertion measured using reliable and valid tools for both groups.

Studies also need to be sufficiently powered, with many acute heading trials having either small sample sizes [19, 20] or high heterogeneity between participants in terms of age/years of experience or sex of players [1]. In an earlier systematic review and meta-analysis on the effects of heading, eight out of 37 studies (22%) had insufficient data to enable effect size calculations [20]. From the remaining 29 studies, 16 studies explored the acute effect of heading, with nine studies having sample sizes with fewer than 32 participants (mean 54 years; range 10–240 years) despite most studies using an experimental research design [20].

## Procedure to Determine Recommendations for Quality Criteria

The members of the Expert Panel were convened to draw on expertise from a range of countries and football associations as well as considering diversity in professional backgrounds. Members of the group included: Kerry Peek (KP, Australia), Martino Franchi (MVF, Italy), Koen Lemmink (KL, The Netherlands), Paul Balsom (PB, Sweden) and Tim Meyer (TM, Chair, Germany). The members were chosen based on expertise in heading research (KP, TM), exercise physiology and biomechanical expertise (MVF), physiological expertise in football research (KL) and involvement in policy making for header training (KP, PB, TM). Members were also selected to be representative of UEFA member associations in covering not only countries with similar heading regulations.

The first step in the discussion on determining minimum quality criteria for acute heading research was to screen existing heading research. Knowledge was gained from a recent literature review led by KP (with co-authors from Football Australia and TM) [14] as well as a literature search on the short-term, medium-term, and long-term effects of heading completed on behalf of UEFA. This search led to the retrieval of references from database searching as well as the reference lists of the included studies. The search terms employed were deliberately broad (“football” or “soccer” and “head*”) to capture as many studies as possible. The Appendix in the Electronic Supplementary Material details the acute heading studies identified through the literature review process.

The second step consisted of identifying core design decisions made by authors of the retrieved studies when designing and conducting their research on the acute effects of heading. The specific methodological elements were extracted from the retrieved studies by KP and TM. These elements were then reviewed, refined and agreed upon by the entire panel.

The third step, prepared by KP and TM, included a formulation of methodological requirements for the identified design decisions. Two rounds of reviews led to a final agreement on the list of quality criteria that will be applied to the next round of UEFA grant applications (Table 1).

**Table 1:** Quality criteria to be applied by the Union of European Football Associations when considering funding for acute heading research

**Questions that will be considered to assess quality criteria**
**Participants**
*Is a control group/comparator condition used?*
*Does the control group/condition simulate a heading movement?*
*Is the control group matched with the intervention group (e.g. age, sex, experience)?*
*Has heading experience and exposure (including age of first exposure) of all participants been reported?*
*Will history of concussion/s be considered and reported?*
*Will playing level and experience of football participants be considered and reported?*
** Heading trials**
*Is the heading trial representative of match play or football practice? A reference has to be given to justify ‘header dosage’*
*Is a ball machine (or equivalent) used to ensure consistent speed, distance and direction?*
*Will forces to the head be reported using 6 degrees of freedom (based on anatomical considerations)?*
* Is there any evidence that the measured “external” forces on the head validly describe the forces experienced by the brain?*
**Confounding variables**
*Will all confounding variables be sufficiently considered and reported to enable an assessment of their influence on the results?*
** Statistics**
*Are appropriate statistical analyses being proposed including the reporting of effect sizes and 95% confidence intervals instead of only p-values?*
*Is there a sample size calculation and power analysis?*
*What considerations have been made in the case of low sample sizes?*
*Are all tests reported with considerations of corrections for multiple testing (related target variables)?*
*Will sub-group analyses be performed (i.e. for sex or age group)?*
*Will the results be considered in the context of clinical impairment?*
**Dependent/target variables and their measurement**
*Will the study use established tests or, alternatively, adequately demonstrate validity of all tests being used?*
*Are the timepoints of measurements considered to adequately capture recovery time where changes to outcome measures from baseline scores are demonstrated post-heading trials or control condition?*
*Has test familiarisation been considered to reduce the effect of repetition alone?*
*Is head acceleration being measured during all conditions (or only with heading trials)?*
*Are blood or other biomarkers being used (and will these be compared to other ‘active’ conditions)?*

## Explanations for the Chosen Quality Criteria

A key design necessity for acute heading studies is that studies should have good external validity rather than focussing on a high likelihood of “significant results”. This is underpinned by a safety concern that participants should not be exposed to unnecessary risk. This is particularly important because if the heading trial protocol does not reflect football practice or match play, then the results will lack external validity and could be misleading. Studies aiming to maximise the contrast (particularly for mechanistic research) should clearly state this and consider the ecological validity of the study when interpreting results. Table 1 demonstrates the minimum criteria agreed by the Expert Panel for acute heading studies, which can be applied to the broader heading research literature but certainly should be considered when drafting project proposals that seek UEFA funding. These criteria will be applied in addition to general aspects of good study design, which are important for any study irrespective of the topic area (such as The Strengthening the Reporting of Observational Studies in Epidemiology [STROBE]) [21].

### Participants

The recruitment strategy should be designed to minimise selection bias and to ensure that the study participants are representative of the target population particularly where participants ‘self-select’ to participate [22]. For instance, one option could be to recruit football players from the same team or club with participation rates collected and reported as well as any reasons for non-participation considered when interpreting the results [22].

#### Control Group/Comparator Condition

Studies that have reported their findings based on one group of participants who complete heading trials in the absence of a control group or comparator condition are problematic for a few reasons. Most importantly, for one group of pre-study and post-study designs, it is almost impossible to determine whether any effects can be attributable to heading only. Cross-over designs are the preferable study design method where the same group of players are exposed to heading trials as well as another one or two conditions, such as a simulated heading (where the participant pretends to head a ball but no actual ball-head impact occurs) or other exertional activity (such as kicking a ball). If using a cross-over study design, it is also vital that the order in which each individual player completes each condition is randomised to reduce the potential confounding effects of fatigue [1].

Where a control group of participants is employed, it is important that the intervention and control participants are matched in terms of age, sex, playing position, level and experience as well as being similar with respect to other confounding factors and the careful consideration of exclusion criteria such as a history of concussion and co-existing conditions such as mental health disorders or epilepsy. Consideration should be given to exclusion of participants with no or limited experience of heading unless this is part of the aim of the study (which should be clearly stated). Similarly, goal keepers, a position shown to infrequently head the ball [23], should be either matched between groups or excluded altogether. Ideally, heading studies should employ equal numbers of male and female participants with a priori sample sizes calculated to allow adequately powered sub-group analyses by sex.

### Heading Trials

As emphasised earlier, justification should be provided by researchers on how and why the ‘header dosage’ as well as the time interval between headers were determined and should be representative of match play or football practice and supported by appropriate references, given that an increasing number of studies are now available to facilitate such a procedure [2, 7, 23–26]. The same criterion applies to ball delivery speeds, which should be calculated for all studies regardless of whether a ball launching machine is used or the ball is thrown. Consistency between each ball delivery must be demonstrated using an angle of delivery that is ecologically valid (e.g. balls dropped from a height directly above the player, while consistent in delivery speed and distance, will not reproduce the flight of a kicked or thrown ball) [1].

Further, it is recommended that forces to the head should be reported using 6 degrees of freedom (based on anatomical considerations) as any force to the head (even linear force) will lead to some rotational forces on the brain. Some discussion also needs to be provided on how any external force measurements (such as head acceleration) translate to internal forces within the head or brain (even if this is hypothesised) and supported by appropriate references (where the evidence does not currently exist).

### Confounding Variables

It is important that any potential confounding variables are sufficiently considered with a view to including them within the analysis and reporting of results to enable an assessment of their influence. Confounding and relevant variables might include: concussion history, concussion symptoms, neurological disorders and history of head, neck or facial injury, consumption of alcohol and drugs, mental illness, player position (goalkeeper specifically), heading exposure (given that heading exposure will vary greatly between players even with a similar length of playing career and experience), and age of first exposure to heading, skill level and football experience, participation in other contact or collision sports (such as rugby or American Football) and neck muscle properties [27, 28]. Where possible, an objective valid measurement of confounding variables should be used, with limited use of self-reported measures (unless this is the only option).

### Statistics

In addition to the reporting of a priori sample size calculation to determine whether the study was adequately powered for the primary outcome measure, it is also expected that study methodology conforms to study design quality criteria such as STROBE [21] or the Consolidated Standards of Reporting Trials (CONSORT) statement extended for cross-over trials [29]. This includes the use of appropriate statistical analyses based on study design and outcome measures including the reporting of effect sizes and 95% confidence intervals instead of only *p*-values. It is also important that all statistical tests are reported, preferably with access to data via an accessible research data repository. Sub-group analyses should also be considered for appropriate groups of participants such as by age or sex. To the authors' knowledge, no study in this area has been published to date with the utmost transparency as a registered report.

### Dependent/Target Variables and Their Measurement

Four categories of target variables broadly exist for heading studies: structural (e.g. magnetic resonance imaging), functional (e.g. postural control, balance, biomarkers), cognitive (e.g. verbal learning, working memory, executive function, psychomotor speed, attention) and behavioural (e.g. impulse, emotional changes).

Researchers should give preference to the selection of appropriate tools with established reliability and validity. For instance, the International Traumatic Brain Injury Common Data Elements Project aims to standardise definitions and protocols for traumatic brain injury research, and information provided on reliable and valid outcome measures might also be applicable to studies exploring the acute effects of heading on brain function [30]. Where such a tool does not currently exist, researchers should undertake to demonstrate reliability and validity themselves before applying these tools in their acute heading study.

Careful consideration should also be given to the longitudinal collection of data even for acute effects. If an effect is demonstrated immediately after completing heading trials, the time taken to return to baseline is an important contribution to the literature. Measurements taken at regular intervals or on consecutive days without further interventions (or head impacts) are preferred and may have a larger influence.

Test familiarisation might be necessary to reduce the effect of repetition alone and, therefore, the number of familiarisation tests required to achieve a stable score for each measure [31] will need to be considered in the research protocol, with appropriate pilot testing of measures before the study commences. This may be particularly important for heading studies in a youth football population.

If head acceleration is to be measured during the heading trials, then this measurement should also be applied to the other active control conditions (such as simulated heading or a kicking condition). Likewise for any blood biomarkers (e.g. S100B), determination of these levels in comparison to other active conditions should be considered.

## Conclusions

The Expert Panel recommends minimum standards for research that explore the acute effects of heading. Studies should consider the use of a cross-over design, or if a control group is used, participants should be matched for age, sex, position, playing level and football experience, while carefully considering exclusion criteria. Consistency in ball size/mass/pressure, ball delivery, angle and speeds as well as accurate measurement of head impact magnitude are important, using tools that measure using 6 degrees of freedom. Appropriate statistical analyses with reporting of effect sizes (with confidence intervals) and sub-group analyses are expected. The potential target variables of structural, functional and cognitive effects of heading should be measured using established tools with appropriate longitudinal data collection. By applying minimum standards for research that explore the acute effects of heading, it is hoped the quality of heading research will be improved and the evidence base to support any potential changes to heading policy or practice will be grounded in high-quality evidence.

### Supplementary Information

Below is the link to the electronic supplementary material.

Supplementary file1 (DOCX 111 KB)
