# Supplementary material for: Quality Criteria for Studies Assessing the Acute Effects of Heading: Results from a UEFA Expert Panel
Source: Sports Med. 2023 Dec 27;54(5):1089–95. doi: 10.1007/s40279-023-01977-z (PMC11127840; doi:10.1007/s40279-023-01977-z)
Supplement: Supplementary file 1 — Supplementary file1 (DOCX 111 KB) [file 40279_2023_1977_MOESM1_ESM.docx]

**Appendix I- List of included ‘acute’ heading studies in the methodological review.**

1. Ashton, J., Coyles, G., Malone, J. J., & Roberts, J. W. (2021). Immediate effects of an acute bout of repeated soccer heading on cognitive performance. *Science and Medicine in Football,* 5(3), 181-187.
2. Bamac¸, B., Tamer, G.S., Colak, T., Colak, E., Seyrek, E., Duman, C., Colak, S., and O¨ zbek, A. (2011). Effects of repeatedly heading a soccer ball on serum levels of two neurotrophic factors of brain tissue, BDNF and NGF, in professional soccer players. *Biol. Sport* 28, 177– 181.
3. Broglio SP, Guskiewicz KM, Sell TC, et al. No acute changes in postural control after soccer heading. *British Journal of Sports Medicine* 2004;38(5):561-67.
4. Caccese, J. B., Buckley, T. A., Tierney, R. T., Rose, W. C., Glutting, J. J., & Kaminski, T. W. (2021). Postural control deficits after repetitive soccer heading. *Clinical Journal of Sport Medicine*, 31(3), 266-272.
5. Cayuela, A. C., Vila, S. A., & Casas-Baroy, J. C. (2020). Frontal lobe executive dysfunction in short-term attentional control following a header in women’s soccer. *Campa d,* 176.
6. Di Virgilio, T.G., Hunter, A., Wilson, L., Stewart, W., Goodall, S., Howatson, G., Donaldson, D.I., and Ietswaart, M. (2016). Evidence for acute electrophysiological and cognitive changes following routine soccer heading. *EBioMedicine* 13, 66–71.
7. Dorminy, M., Hoogeveen, A., Tierney, R.T., Higgins, M., McDevitt, J.K., and Kretzschmar, J. (2015). Effect of soccer heading ball speed on S100B, sideline concussion assessments and head impact kinematics. *Brain Injury*. 29, 1158–1164.
8. Gallant, C., Drumheller, A., & McKelvie, S. J. (2017). Effect of improper soccer heading on serial reaction time task performance. *Current Psychology,* 36, 286-296.
9. Gutierrez, G.M., Conte, C., and Lightbourne, K. (2014). The relationship between impact force, neck strength, and neurocognitive performance in soccer heading in adolescent females. *Pediatic Exercise Science.* 26, 33–40
10. Haran, F. J., Tierney, R., Wright, W. G., Keshner, E., & Silter, M. (2013). Acute changes in postural control after soccer heading. *International Journal of Sports Medicine,* 34(04), 350-354.
11. Harriss, A.B., Abbott, K., Kimpinski, K., Holmes, J.D., Johnson, A.M., Walton, D.M., and Dickey, J.P. (2019). An evaluation of heart rate variability in female youth soccer players following soccer heading: a pilot study. *Sports* 7, 229.
12. Huibregtse, M. E., Nowak, M. K., Kim, J. E., Kalbfell, R. M., Koppineni, A., Ejima, K., & Kawata, K. (2020). Does acute soccer heading cause an increase in plasma S100B? A randomized controlled trial. *Plos one*, *15*(10), e0239507.
13. Hwang, S., Ma, L., Kawata, K., Tierney, R., & Jeka, J. J. (2017). Vestibular dysfunction after subconcussive head impact. *Journal of Neurotrauma,* 34(1), 8-15.
14. Kaminski, T.W., Thompson, A., Wahlquist, V.E., and Glutting, J. (2020). Self-reported head injury symptoms exacerbated in those with previous concussions following an acute bout of purposeful soccer heading. *Research in Sports Medicine.* 28, 217–230.
15. Kawata, K., Tierney, R., Phillips, J., and Jeka, J.J. (2016). Effect of repetitive sub-concussive head impacts on ocular near point of convergence. *International Journal of Sports Medicine,* 37, 405–410.
16. Kenny, R.A., Mayo, C.D., Kennedy, S., Varga, A.A., Stuart-Hill, L., Garcia-Barrera, M.A., McQuarrie, A., Christie, B.R., and Gawryluk, J.R. (2019). A pilot study of diffusion tensor imaging metrics and cognitive performance pre and post repetitive, intentional sub-concussive heading in soccer practice. Journal of Concussion 3, 2059700219885503
17. Mangus, B. C., Wallmann, H. W., & Ledford, M. (2004). Soccer: Analysis of postural stability in collegiate soccer players before and after an acute bout of heading multiple Soccer balls. *Sports biomechanics*, 3(2), 209-220.
18. Nowak, M. K., Bevilacqua, Z. W., Ejima, K., Huibregtse, M. E., Chen, Z., Mickleborough, T. D., ... & Kawata, K. (2020). Neuro-ophthalmologic response to repetitive subconcussive head impacts: a randomized clinical trial. *JAMA ophthalmology,* 138(4), 350-357.
19. Putukian, M., Echemendia, R. J., and Mackin, S. (2000). The acute neuropsychological effects of heading in soccer: a pilot study. *Clinical Journal in Sports Medicine.* 10, 104–109.
20. Rieder, C., and Jansen, P. (2011). No neuropsychological consequence in male and female soccer players after a short heading training. *Archives Clinical Neuropsychology.* 26, 583–591.
21. Schmitt, D. M., Hertel, J., Evans, T. A., Olmsted, L. C., & Putukian, M. (2004). Effect of an acute bout of soccer heading on postural control and self-reported concussion symptoms. *International Journal of Sports Medicine,* 25(05), 326-331.
22. Stalnacke, B.M., and Sojka, P. (2008). Repeatedly heading a soccer ball does not increase serum levels of S-100B, a biochemical marker of brain tissue damage: an experimental study. *Biomark. Insights* 3, 87–91.
23. Wallace, C., Smirl, J.D., Zetterberg, H., Blennow, K., Bryk, K., Burma, J., Dierijck, J., Wright, A.D., and Van Donkelaar, P. (2018). Heading in soccer increases serum neurofilament light protein and SCAT3 symptom metrics. *BMJ Open Sport & Exercise Medicine.* 4, e000433.
24. Wirsching, A., Chen, Z., Bevilacqua, Z.W., Huibregtse, M.E., and Kawata, K. (2019). Association of acute increase in plasma neurofilament light with repetitive subconcussive head impacts: a pilot randomized control trial. *Journal of Neurotrauma.* 36, 548–553.
25. Zetterberg, H., Jonsson, M., Rasulzada, A., Popa, C., Styrud, E., Hietala, M.A., Rosengren, L., Wallin, A., and Blennow, K. (2007). No neurochemical evidence for brain injury caused by heading in soccer. *British Journal of Sports Medicine*. 41, 574–577.
